# Preparation of uniformly labelled ^13^C- and ^15^N-plants using customised growth chambers

**DOI:** 10.1186/s13007-020-00590-9

**Published:** 2020-04-06

**Authors:** Asja Ćeranić, Maria Doppler, Christoph Büschl, Alexandra Parich, Kangkang Xu, Andrea Koutnik, Hermann Bürstmayr, Marc Lemmens, Rainer Schuhmacher

**Affiliations:** 1 Institute of Bioanalytics and Agro-Metabolomics, Department of Agrobiotechnology (IFA-Tulln), University of Natural Resources and Life Sciences Vienna (BOKU), Konrad-Lorenz-Straße 20, 3430 Tulln, Austria; 2 Institute of Biotechnology in Plant Production, Department of Agrobiotechnology (IFA-Tulln), University of Natural Resources and Life Sciences Vienna (BOKU), 3430 Tulln, Austria

**Keywords:** ^13^CO_2_ atmosphere, ^15^N-containing nutrient solution, Stable isotopic labelling, Internal standard, Metabolomics, Cultivation of wheat, LC-HRMS

## Abstract

**Background:**

Stable isotopically labelled organisms have found wide application in life science research including plant physiology, plant stress and defense as well as metabolism related sciences. Therefore, the reproducible production of plant material enriched with stable isotopes such as ^13^C and ^15^N is of considerable interest. A high degree of enrichment (> 96 atom %) with a uniformly distributed isotope (global labelling) is accomplished by a continuous substrate supply during plant growth/cultivation. In the case of plants, ^13^C-labelling can be achieved by growth in ^13^CO_2(g)_ atmosphere while global ^15^N-labelling needs ^15^N- containing salts in the watering/nutrient solution. Here, we present a method for the preparation of ^13^C and ^15^N-labelled plants by the use of closed growth chambers and hydroponic nutrient supply. The method is exemplified with durum wheat.

**Results:**

In total, 330 g of globally ^13^C- and 295 g of ^15^N-labelled *Triticum durum* wheat was produced during 87 cultivation days. For this, a total of 3.88 mol of ^13^CO_2(g)_ and 58 mmol of ^15^N were consumed. The degree of enrichment was determined by LC-HRMS and ranged between 96 and 98 atom % for ^13^C and 95–99 atom % for ^15^N, respectively. Additionally, the isotopically labelled plant extracts were successfully used for metabolome-wide internal standardisation of native *T.durum* plants. Application of an isotope-assisted LC-HRMS workflow enabled the detection of 652 truly wheat-derived metabolites out of which 143 contain N.

**Conclusion:**

A reproducible cultivation which makes use of climate chambers and hydroponics was successfully adapted to produce highly enriched, uniformly ^13^C- and ^15^N-labelled wheat. The obtained plant material is suitable to be used in all kinds of isotope-assisted research. The described technical equipment and protocol can easily be applied to other plants to produce ^13^C-enriched biological samples when the necessary specific adaptations e.g. temperature and light regime, as well as nutrient supply are considered. Additionally, the ^15^N-labelling method can also be carried out under regular glasshouse conditions without the need for customised atmosphere.

## Background

The use of plants and plant-derived metabolites labelled with heavy stable isotopes offers an interesting perspective in phytochemical research. Some research fields where stable isotopes found application are nutrition [1], evaluation of biosynthetic pathways [2], metabolic engineering [3], flux analysis [2, 4], accurate quantification of plant metabolites [5], studies on plant–microbe interactions [6] and untargeted metabolomics and proteomics [6–12]. The increasing popularity of isotopically labelled plant material goes hand in hand with the development of complementary analytical tools e.g. NMR and high throughput GC–MS and LC-HRMS which enable differentiation between native and labelled metabolites [13, 14] as well as the availability of bioinformatics (for automated data processing) and statistical tools (for differential metabolome analysis). Therefore, protocols for the reproducible production of globally labelled biological organisms including higher plants with target stable isotopes are of increasing interest.

Up to now, a variety of in vivo stable isotopic labelling methodologies for different organisms were described (e.g. organelles, cells, tissue/organs and whole plants) [3, 15, 16]. These approaches are based on the application of isotopically labelled substrates to the organism under investigation which further converts the labelled substrate to various downstream metabolites via endogenous enzymes. Depending on the labelling regime, the organism can be labelled uniformly or non-uniformly with different degrees of enrichment. In tracer [2, 17] or pulse-based methods (time-dependent substrate exposure) [4, 18] a specific submetabolome is labelled. These study types aim to elucidate metabolic networks or determine the rates of metabolic transformations or enzymatic reactions. On the other side, globally labelled organisms can be used for metabolome- or proteome-wide internal standardisation in untargeted -omics approaches which aim to capture the total number of the respective biochemical constituents. In addition, metabolic alterations which may, for example, be caused by various stress factors can be comprehensively investigated.

Global ^13^C-labelling is used to produce uniformly ^13^C-labelled plants (here termed as ^13^C^14^N plants) and requires a continuous supply of ^13^CO_2_ throughout the whole cultivation. The ^13^CO_2_ is applied as a substrate which is *in planta* further converted to all assimilates required to sustain plant metabolism. Therefore, airtight cultivation chambers (labelboxes) with ^13^CO_2_ atmosphere become necessary. As the preparation of ^13^C-enriched plants affords long-term cultivations the technical equipment has to enable the regulation of growth parameters such as temperature, light and the atmospheric humidity as well as the levels of CO_2_ and O_2_. Global ^15^N-labelling is accomplished by applying ^15^N-containing nutrient solution through the root system and ^15^N distribution takes place by transport via xylem and the initial formation of ^15^N-labelled glutamine in the plastids and further via transamination reactions. In such a way, ^15^N-labelled plants (termed ^12^C^15^N) can be produced. As ^15^N is applied via the nutrient solution, hydroponic cultivation with a ^15^N-labelled ammonium- and/or nitrate solution are prerequisite for defined labelling experiments. If both labelling regimes are used/applied in parallel under the same experimental conditions (cultivation chamber and hydroponics), the obtained plants can be expected to have similar metabolic composition and their metabolomes (^13^C^14^N and ^12^C^15^N) can be correlated. Alternatively, if only ^15^N-labelling is required the ^15^N salts can be also provided without the use of cultivation chambers.

Up to know, the stable isotopically labelling procedures are implemented in the labs of other research groups who were successful at producing different plants with different degrees of enrichment with ^13^C [18–22], ^15^N [23–26] and using both elements in form of dual labelling (^13^C^15^N plants) or single labelling of plants cultivated in parallel (^13^C^14^N and ^12^C^15^N plants) [13, 15, 16, 27]. Here, we aim to present a fully automated and robust labelling equipment implemented to produce highly ^13^C- and ^15^N-enriched wheat, cultivated in parallel under the same conditions with high N and C metabolome coverage. The presented procedure describes the detailed setup and experimental instructions for the automated long-term cultivation of wheat plants allowing plant manipulation/treatment in closed atmosphere. The procedure enables the production of plants highly enriched with ^13^C (> 96 atom %) via the ^13^CO_2_ atmosphere whereas the CO_2_ concentration can be controlled during the cultivation. The moisture levels can be limited and CO_2_ or ethylene can be removed by the optional application of a scrubbing device. The included irrigation system allows introduction of nutrient solution into the closed atmosphere. Additionally, ^15^N-labelling can be performed by enrichment of the nutrient solution with ^15^N-containing salts which makes it possible to produce up to 99 atom % of ^15^N in ^12^C^15^N plants. A dual labelling combining ^13^C and ^15^N is also possible. The described setup can easily be extended/adjusted to cultivate other highly ^13^C-enriched uniformly labelled plants. Moreover, the presented labelbox setup also allows pulse labelling experiments to study metabolic processes.

## Results and discussion

The presented method describes the cultivation of *T. durum* plants from seedling to flowering stage, for the production of ^13^C^14^N plants under controlled ^13^CO_2_ atmosphere and ^12^C^15^N material by supply with ^15^N-labelled NH_4_NO_3_, Ca(NO_3_)_2_, and KNO_3_ containing nutrient solution. To demonstrate both cultivation and the exemplary application of the plant material in an untargeted metabolomics approach, the paper is structured as follows: (a) cultivation in the labelbox for the production of ^13^C^14^N and ^12^C^15^N *T.durum* including detailed documentation of the applied and recorded parameters; (b) enrichment of the generated biomass of wheat and the consumption of CO_2_ and nutrient solution; (c) assessment of the ^13^C and ^15^N enrichment by high resolution mass spectrometry and (d) application of the labelled wheat in an isotope assisted untargeted LC-HRMS approach with the aim to elucidate the ^13^C and/or ^15^N-containing metabolome. Additionally, some novel aspects as well as comparison with already presented protocols for stable isotopically labelling will be discussed.

### Wheat cultivation in the labelbox

**Fig. 1:**
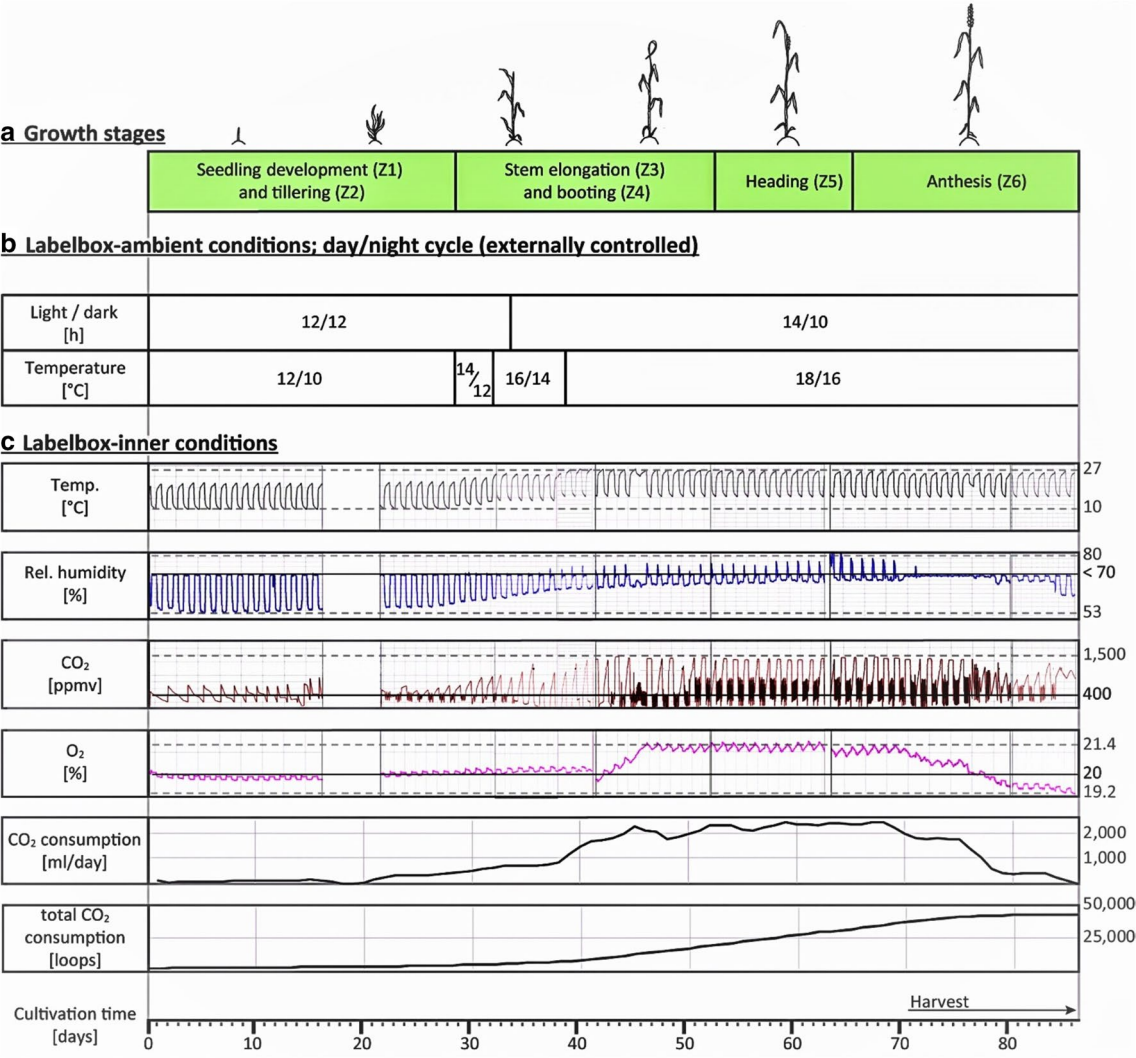
^13^C-labelbox cultivation record of wheat for the inner atmosphere correlated to the observed growth stages and applied setpoint levels for ambient temperature and light duration. Similar records were obtained for the ^15^N-labelbox

The cultivation of 4 wheat plants in each labelbox lasted 87 days in total. Defined temperature and light regimes were applied depending on the growth stage. The atmospheric parameters inside the labelbox (inner temperature, humidity as well as CO_2_ and O_2_ levels) were recorded in regular time-intervals (shown in Fig. 1) over the whole cultivation period.

The temperature and light regime used during cultivation was adapted to 24 h cycles to simulate natural day and night rhythm for summer wheat. Therefore, the ambient temperature (outside the label box) was increased stepwise while the light exposure was extended in the course of cultivation. To simulate day time conditions, the plants were exposed to artificial light during the first 34 days for 12 h, while from day 35 the exposure was extended by 2 h (14 h/10 h at day/night). The ambient temperature setpoints were increased during the cultivation in three steps (Fig. 1b). In parallel, the temperature inside the labelbox was recorded with the integrated temperature sensor. The inner temperature reached ambient conditions during night, while it was up to 10 °C higher than that of the ambient climate chamber during the day, due to the energy provided by the external light source, regardless of the time point during cultivation (Fig. 1c).

The first two monitored growth stages were (Z1) seedling development and (Z2) tillering (Fig. 6a, b). During this period, which represents about one-third of the cultivation time, the temperature inside the labelbox was 10 °C during night and 22 °C during the day (Fig. 1c) resulting in 3 to 6 tillers per plant. During that stage, the daily CO_2_ consumption was between 50 and 100 ml (25 to 50 loops) of CO_2_ per day and CO_2_ levels did not increase during night (Fig. 1c). Further, during the day the O_2_ levels were slightly below 20% while the relative humidity was recorded to be 53%. During the night intervals, Peltier cooling was necessary to keep relative humidity at the maximum allowed 70% which depicts the higher humidity through the release of H_2_O as plant transpiration product via the stomata.

The first temperature increase initiated the stem elongation stage (Z3) (Fig. 6c). The resulting increase in biomass is reflected by an elevated CO_2_ consumption during the day and higher CO_2_ levels in the dark (up to 1500 ppmv). CO_2_ was not removed from the atmosphere in the labelbox as it was quickly assimilated after starting the day time period and this helped to save expensive ^13^CO_2_. The O_2_ level slightly increased in comparison to the Z1/Z2 stage but was still below 21%. High O_2_ levels would reduce the rate of photosynthesis as shown in [28]. It was also observed that the increase in biomass and the associated elevated transpiration and respiration steadily raised relative humidity levels during the day to reach ca. 64% at day 40 (Fig. 1c). Similar records were obtained in the ^15^N-labelbox.

During growth stages Z4–6 (corresponding to booting, heading and anthesis), the recorded parameter levels were maintained at setpoint levels. O_2_ levels and relative humidity reached and stayed at max. values with ~ 21% and 70%, respectively. With the biomass, the increase in CO_2_ consumption was further observed to rise further up to ~ 2.3 l (1100 loops) at day 66. High demand on CO_2_ can also be seen from short intervals between dosages to keep CO_2_ at an average level of 400 ppmv (Fig. 1c).

The harvest period lasted a total of 14 days. Approximately 6 days (144 h) after first anthers appeared, the respective ear and the adjacent stem and leaves were cut, removed from the box and frozen in liquid nitrogen. As expected, the removal of plant material from the labelbox resulted in a decrease of CO_2_ consumption, O_2_ levels and relative humidity (Fig. 1c), starting from day 71.

Summarised, the recorded cultivation conditions are in accordance with the applied temperature- and light regimes and also reflect both the increase in biomass as well as photosynthesis and respiration activity during growth of the plants in the labelbox.

### Yield of biomass and consumption of ^13^CO_2_ and ^15^N-enriched nutrient solution

**Fig. 2:**
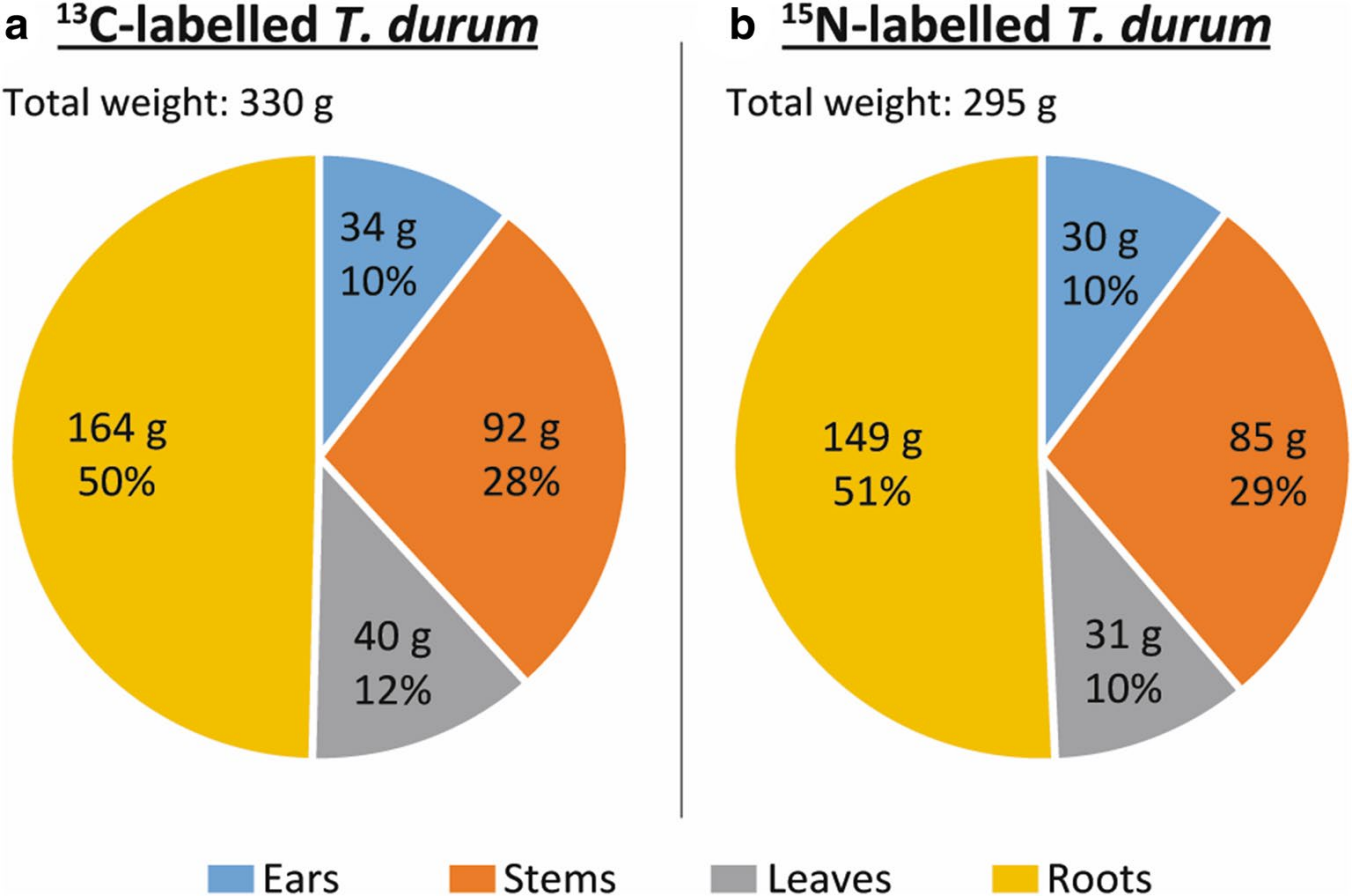
Mass fractions in [%] relative to the whole wheat plant weight

In total, 330 g of ^13^C^14^N- and 295 g of ^12^C^15^N-labelled plant fresh weight (FW) were harvested from two labelboxes. The mass fractions obtained for different plant organs was similar for both labelboxes. Roots represented the greatest mass fraction (50%), followed by stems (ca. 30%), leaves and ears (ca. 10% each) (Fig. 2).

The total water content in FW was determined to be between 64% and 70% for different wheat organs. After freeze drying, the rest moisture levels of the plant organs were below 5%. The consumption rate of the nutrient solution in the ^15^N-labelled wheat was 24.4 ml/g FW and 870 mg of ^15^N was consumed in total. In the ^13^C-experiment, 3.81 mol of ^13^CO_2_ were consumed which corresponds to a consumption rate of 11.5 mmol ^13^CO_2_/g FW (Additional file 1: Equation (S1, S4)). The consumed amounts of ^15^N or ^13^CO_2_ are in good agreement with the literature [29–31].

### Determination of the degree of ^13^C- and ^15^N enrichment by LC-HRMS

**Fig. 3:**
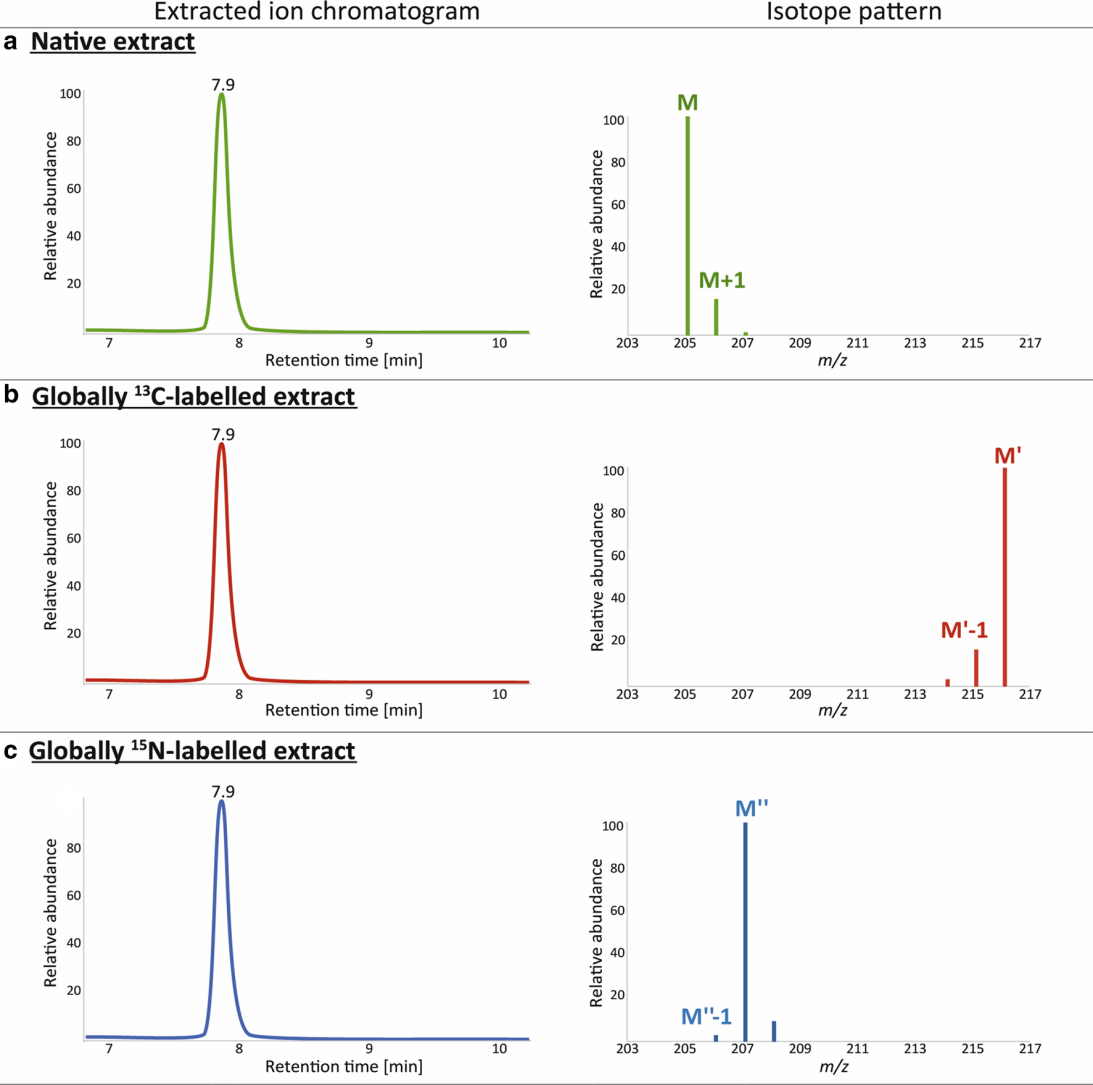
In each experiment high abundant isotopologues of tryptophan were represented as M (all carbons are ^12^C), M’ (all carbons are ^13^C) and M’’ (all nitrogens are ^15^N) and the low abundant isotopologues of the same molecule are M + 1 (one ^13^C and rest ^12^C), M’-1 (one ^12^C and rest ^13^C) and M’’-1 (one ^14^N and rest ^15^N). The relative isotopologue intensities were used to determine the degree of enrichment

The degree of isotopic enrichment with ^13^C and ^15^N, were determined by LC-HRMS in ^13^C^14^N and ^12^C^15^N wheat ear extracts respectively. For this, the LC-HRMS data was firstly processed by the MetExtractII software [14]. Then, ca. 9 highly abundant metabolites from the obtained list that contains truly wheat-derived metabolites were selected and used to determine the degree of enrichment. A detailed annotation or identification was beyond of the scope of this study. However, as ^13^C- and ^15^N-labelling was employed the number of C and N atoms contained in the target compounds were known exactly. The considered metabolites contained between 9 and 14 carbon atoms and 1 to 4 nitrogen atoms, respectively. Further, the isotopologue abundance distribution for each metabolite in measured mass spectra were examined manually with Xcalibur 4.0 (Thermo Fisher Scientific, Bremen, Germany) and exemplified with one tested compound (tryptophan) in Fig. 3. All isotopologues of the same metabolite show the same retention times as they coelute from the chromatographic column.

For calculation of the degree of enrichment, peak height intensities at three different regions of chromatographic peak (centre, front and tail of peak) were considered. MS peak height intensities > 10^6^ were taken into account to accurately determine the degree of isotopic enrichment [32].

The degree of enrichment (E_X_) for native and labelled forms was calculated according to Eq. (1) which is derived from the binominal distribution equation [33]. R_X_ represents the intensity ratio of the inspected isotopologues of a molecule. The count _C/N_ is the number of carbon or nitrogen atoms contained in the molecule. The I_M+0.997_ represents the intensity of the low abundant ^15^N mass peak in native samples.

1 $$ \begin{aligned}& E_{x} \left[ {atom\% } \right] = \left( {1 - \frac{{R_{x} }}{{count_{{{C \mathord{\left/ {\vphantom {C N}} \right. \kern-0pt} N}}} + R_{x} }}} \right);\\ &\quad x = {}^{13}{\text{C}},{}^{15}{\text{N}},{}^{12}{\text{C}}\;or\;{}^{14}{\text{N}}\end{aligned} $$

2 $$ \begin{aligned}& R_{13C} \; = \;\frac{{I_{{M^{\prime} - 1}}}}{{I_{{M^{\prime}}}}};\;R_{15N} \; = \;\frac{{I_{{M^{\prime\prime} - 1}}}}{{I_{{M^{\prime\prime}}}}};\;\\&R_{12C} \; = \;\frac{{I_{M + 1}}}{{I_{M}}};\;R_{14N} \; = \;\frac{{I_{M + \;0.997}}}{{I_{M}}}\end{aligned} $$

The degrees of ^13^C and ^15^N in *T. durum* cultivars were determined to be (98.4 ± 0.1) atom % and (99 ± 0.2) atom %, respectively. Similar levels were obtained for earlier labelling experiments with *T. aestivum* (Table 1). In comparison to other genotypes, the cultivar Apogee had a lower degree of enrichment for ^13^C and ^15^N. This might be explained with the fact that initially native seeds were used. In comparison to other genotypes, the Apogee is a “dwarf wheat”. The amount of produced biological material per seed is ca. 2.8 times lower for Apogee wheat than for Karur plants. Therefore, the fraction of ^12^C and ^14^N per seed that originated from the seeds is higher in Apogee than in the other cultivars.

**Table 1:** Determined degree of enrichment after cultivation of ^13^C- and ^15^N-labelled *T. aestivum* and *T. durum* cultivars as well as the enrichment in native analogues

Higher abundant stable isotope	Sample type	Species	Genotype	Nr. of metabolites	E_x_ [atom %]	Standard deviation [atom %]	Standard error of the mean [atom %]
^13^C	Labelled	*T. aestivum*	Remus	9	98.4	0.1	0.02
			Apogee	9	96.7	0.2	0.05
		*T. durum*	Karur	9	98.4	0.1	0.02
^12^C	Native	*T. aestivum*	Remus	9	98.91	0.75	0.14
			Apogee	9	98.99	0.09	0.02
		*T. durum*	Karur	9	99.00	0.09	0.02
^15^N	Labelled	*T. aestivum*	Remus	8	99.4	0.3	0.05
			Apogee	8	95.6	0.5	0.13
		*T. durum*	Karur	8	99	0.2	0.04
^14^N	Native	*T. aestivum*	Remus	6	99.7	0.1	0.02
			Apogee	7	99.6	0.1	0.02
		*T. durum*	Karur	8	99.7	0.1	0.02

### Annotation of the global metabolome

Extracts of the stable isotopically labelled wheat (^13^C and ^15^N separately) were used to internally standardise native wheat extracts. These sample mixtures were measured with LC-HRMS and processing of the obtained raw data was performed with the in-house developed MetExtract II software [7, 8, 14, 34]. The program automatically performs an efficient filtering of all non-wheat derived metabolites by extracting metabolic features based on their chromatographic coelution and isotopic pattern (which is characteristic to the mixture of native and labelled extracts) [14]. The LC-HRMS data of the ^13^C-experiment contained 1467 ions (global metabolome including all N-containing ions) while 367 ions were found in the ^15^N-experiment (only N-containing ions were considered). In the next step, both feature lists were merged into one matrix by combining ions with same characteristics. For this, *m/z* values and retention times of native isotopologues containing ^12^C and/or ^14^N were considered in both experiments. This combined matrix, encompassed 1621 ions in total. In order to remove false positive ions from the N metabolome (e.g. those containing N but no C), the isotopologue pattern of each metabolite putatively containing N was additionally curated manually by the aid of the MZmine software [35] and the isotopologue pattern was reviewed in all experiments (e.g. in native, ^13^C- and ^15^N-labelling experiment similar isotopologue patterns were expected as those shown in Fig. 3).

**Fig. 4:**
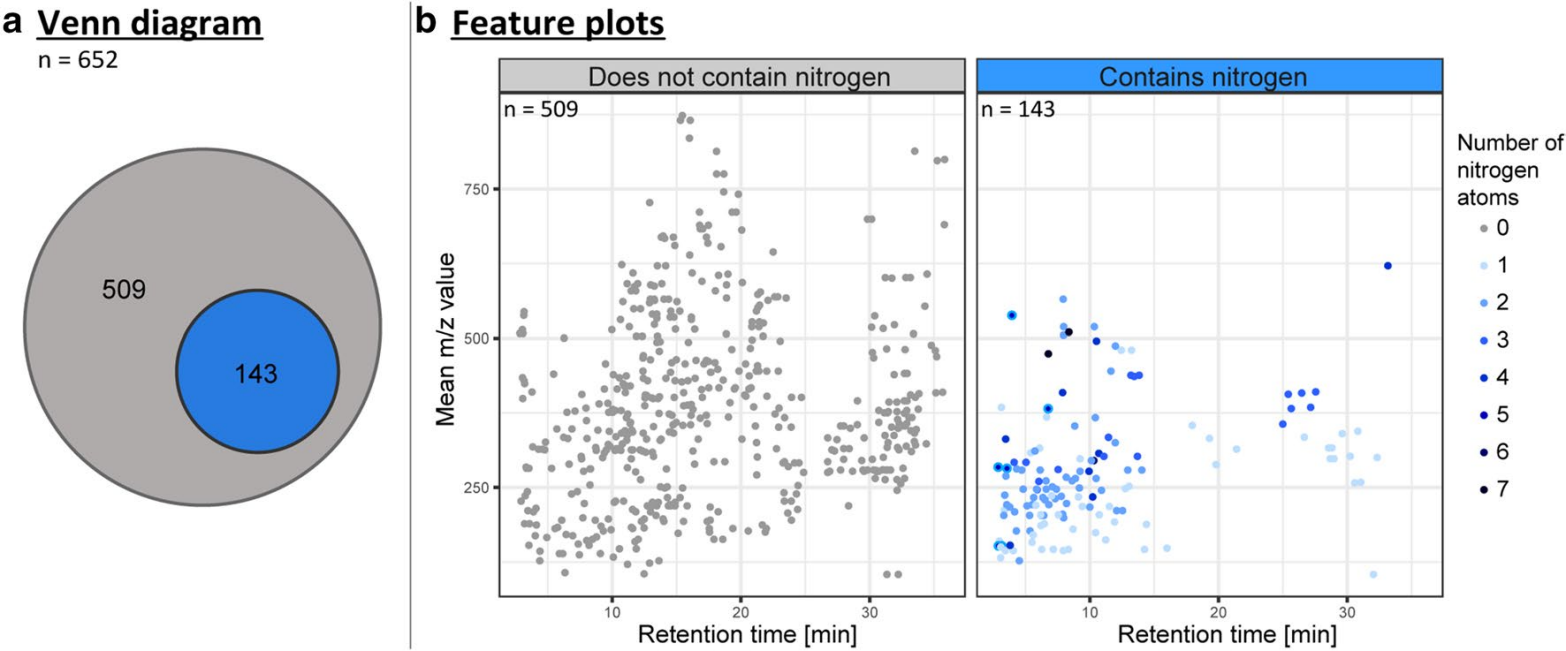
Each dot represents one metabolite found in the ^13^C and ^15^N experiment

Finally, the data set contained 1519 ions which were assigned to 652 unique metabolites with a mass range between *m/z* 116 and 875 (Fig. 4). Amongst the detected metabolites, 143 contained nitrogen. As illustrated in the feature plot of Fig. 4, metabolites containing nitrogen atoms tended to elute during the first 15 min of the chromatographic run. With respect to number, the majority of the detected N-containing metabolites contain 1, 2 or 3 nitrogens.

### Applications of the presented method and comparison with existing protocols

Previously, two types of equipment for stable isotopic labelling of plants with ^13^C were described. The first employs commercially available, smaller sized automatic cultivation chambers with a built-in regulation module for light and temperature regimes [16, 20, 36]. The second one makes use of (in-house designed) labelling devices placed in bigger sized climate chambers, climate rooms or glasshouses which provide an external light and temperature regulation [13, 15, 18, 19, 27]. Both approaches enable continuous monitoring and regulation of atmospheric parameters. Enrichment with ^15^N or ^34^S does not require systems with an enclosed atmosphere but rather hydroponic cultivation systems [23–25, 37]. Generally, both equipment types are in principle suitable to perform pulse [16, 18] and uniformly labelling [13, 20, 38] of plants at different enrichment degrees.

The method described here employs a customised labelbox that can be purchased commercially. In contrast to the existing approaches the presented method enables the manipulation of plants during cultivation by the use of four gloves which are tightly attached to the labelbox. Additionally, a gate compartment with an air lock system constitutes a part of the labelbox, enabling the introduction or removal of manipulation and sampling tools as well as sampled plant material from the labelbox without contaminating the inner atmosphere with ambient ^12^CO_2_ during cultivation.

The presented setup may be also used to mimic abiotic stress—e.g. low or elevated nutrient supply or targeted control of O_2_ or CO_2_ levels [16]. In combination with the application of stable isotopically labelled ^13^CO_2_ the thereby caused molecular alterations may be effectively investigated. Further, the presented equipment is perfectly suited for pulse labelling or—to reduce experimental costs—uniform labelling with lower degrees of enrichment. The latter can be achieved with carbon dioxide showing a lower degree of ^13^C enrichment. In addition, the monitoring of both ^12^CO_2_ and ^13^CO_2_ can be performed in one single box. For pulse labelling, synthetic air consisting of ^14^N_2_ and ^16^O_2_ only might be replaced by CO_2_-containing air and ^13^CO_2_ can be dosed according to the needs of the respective experiment.

While the costs for ^15^N-salts are rather moderate, the ^13^CO_2_ labelling is cost demanding but affordable on a long-run. For example, 330 g of wheat plant material were produced from ~ 100 L ^13^CO_2_ in a single labelbox, giving enough material for global internal standardisation of ~ 10^5^ analytical samples according to the described LC-HRMS workflow. This would correspond to ca. 0.1 euros per sample for global internal standardisation. For making sure that the labelled plant material can be used over a long period, it should be freeze-dried to less than ca. 5% rest moisture and stored at − 80 °C. To our experience, under such conditions, the plant material may be preserved for several years without a significant change of its metabolic composition.

## Conclusion

The method presented in this article enables the reproducible production of stable isotopically labelled plant material under controlled conditions. By the use of a customised growth chamber (labelbox), the composition of the atmosphere inside the labelbox including ^13^CO_2_ can be pre-selected and regulated during long-term cultivation. By this, degrees of global ^13^C enrichment as high as 98% can be achieved. Furthermore, with the presented equipment, plants can also be treated during cultivation without interfering with the labelling process. Complementary ^15^N-labelling is facilitated by watering with ^15^N-enriched nutrient solutions, which can also be done in the greenhouse without the need of the labelbox.

The globally labelled plant material can be used as internal reference in metabolism-related plant research and phytochemical analysis including metabolomics, proteomics and lipidomics [34, 39–42]. The generated plants can also be further processed to generate fractions of tailored substance classes or to isolate pure phytochemicals. Moreover, the labelled plants or generated fractions can be used for internal standardisation of experimental native samples to improve accuracy and reliability in both qualitative and quantitative analysis [43, 44].

While here, method performance characteristics have been evaluated for global uniform ^13^C- and ^15^N-labelling of cereals, the presented approach can also easily be adapted to culture other plant species or used for short-term labelling, the production of partly enriched plant material or kinetic studies using alternate exposure to ^12^CO_2_ and ^13^CO_2_ in pulsed time intervals [18].

## Methods

This chapter includes [1] a description of the technical equipment used for the global ^13^C- and ^15^N-labelling in detail; [2] a presentation of the cultivation procedure adapted to produce globally labelled wheat from seedling to flowering stage and [3] a sample preparation workflow for the applied untargeted LC-HRMS based metabolomics approach.

### Description of the labelling equipment

**Fig. 5:**
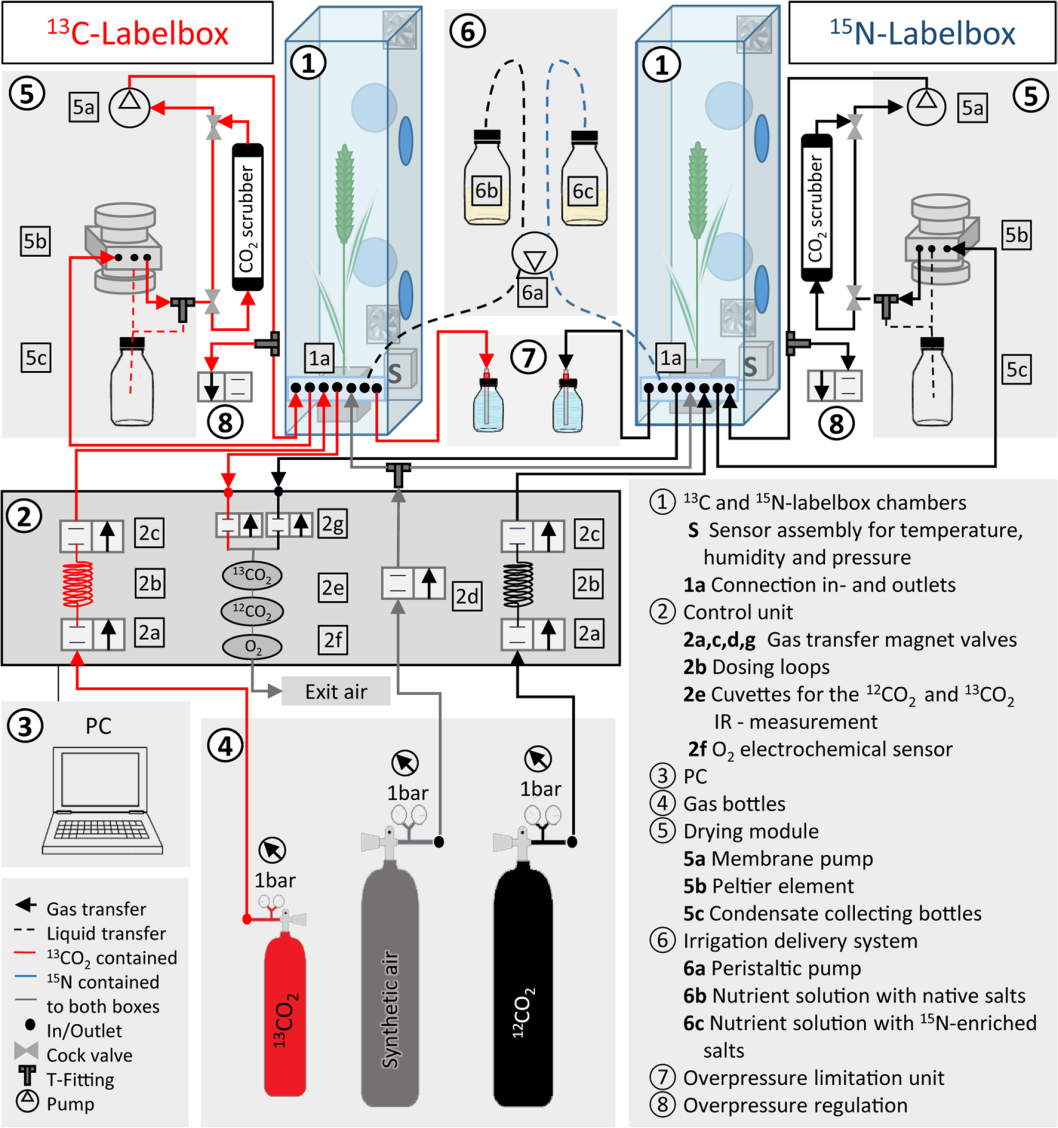
Simplified schematic overview of the labelling process by applying the PhytolabelBox equipment (ECH, Halle Germany)

This section is related to the functional description of the cultivation process to produce globally ^13^C- and ^15^N-labelled plants using the PhytolabelBox equipment (ECH, Halle, Germany). All symbols contained in this section are related to Fig. 5.

The plants were cultivated in two specially designed cultivation chambers, so called labelboxes ①. Each of these 214-L Plexiglas labelboxes is equipped with two internal fans as well as a sensor assembly **S** for temperature, relative humidity and overpressure measurement. Each labelbox also contains 4 openings tightly sealed with gloves (Glove Box Glove Jugitec^®^ H, chlorosulfonated polyethylene, JUNG RUBBERTEC) allowing plant manipulation during cultivation. Control and regulation of cultivation parameters (e.g. CO_2_ supply, overpressure and atmospheric humidity), which are essential to achieve and maintain adequate conditions for plant growth were implemented by different modules, which are together with the labelbox termed as PhytolabelBox equipment. These modules are navigated by the control unit ② based on the user predefined parameter setpoints via the software on the PC ③. Separate from this, the nutrient supply is manually carried over the irrigation delivery module ⑥.

#### Gas supply and control module

CO_2_ is supplied from the gas bottles ④ which are connected via stainless steel and copper lines to the labelbox ①. Since carbon labelling was realised in one of the boxes (^13^C-labelbox), this is supplied with ^13^CO_2_ gas while the ^15^N-labelbox was operated with native ^12^CO_2_. Each gas bottle is equipped with a pressure reducer, which was set to 1 bar. The amount of the introduced CO_2_ is balanced by dosage loops 2b (ca. 2 ml at 1 bar) located in the control unit ②. The dosage loops 2b are covered with magnet valves 2a, c from both sides (in- and outlet). These valves are opened reciprocally in short time intervals during gas dosing operation. The pressure difference between the pressure reducer at the gas bottle and the interior of the labelbox forces the CO_2_ into the loop space and further into the labelbox ①. The CO_2_ [ppmv] measurement was performed by IR absorption using separate cuvettes 2e for ^13^CO_2_ and ^12^CO_2_. Simultaneously, the O_2_ [%] is also measured with an electrochemical sensor 2f, which is located in the control unit. Immediately before measurement, the sensor and cuvettes are rinsed with air from the labelbox to achieve a homogeneous gas sample. The labelbox is always operated at slight overpressure (the difference between ambient and inner pressure) of 10 ± 2 mbar to prevent ambient air from entering the closed and controlled atmosphere.

The overpressure levels are constantly recorded by the integrated sensor S in each labelbox ① and the pressure signal status is continuously transferred via the control unit ② to the PC ③. If the overpressure level decreases below the allowed minimum setpoint (slight gas leaks are expected and occur), the dosing of synthetic air (mixture of N_2_ + O_2_) is activated. To this end, a magnet valve 2d, which is located in the control unit opens and the synthetic air is dosed into the labelbox ① until the overpressure level reaches the setpoint again. If the pressure in the cultivation box exceeds the maximum allowed overpressure of 12 mbar, a magnet valve ⑧ opens to release excess air. This mechanism is controlled by the software and is additionally used for the air exchange in case CO_2_ and/or O_2_ levels exceed the defined maximum values. To prevent damage to the plexiglas labelboxes by excessive overpressure, a power-independent mechanism preventing too high pressure in the closed labelboxes is installed (overpressure limitation unit ⑦). For this, a water-filled bottle connected to the labelbox provides a robust safety tool, as by a simple physical mechanism, overpressure > 15 mbar is automatically released.

#### Humidity control module

To regulate the maximum humidity levels, each cultivation chamber contains an internal relative humidity (polymer-based capacitive) sensor **S**, an external membrane pump 5a and Peltier element 5b as well as a condensate bottle 5c. A permanent gas circulation (30 L/min) is maintained throughout the whole cultivation period passing the Peltier element 5b by use of a membrane pump 5a. In such a way, not only continuous homogenous air is provided to the plants but also relative humidity levels can be limited. The increase of relative humidity above a specified value caused by plant transpiration activates an increase of Peltier current in the drying module. Peltier current forces one side of the block to cool down, which enables water condensation from the air coming from the labelbox. The condensate is further collected in separate bottles whereas the dried air is guided back to the cultivation chambers.

#### Irrigation system

The irrigation system ⑥ consisted of two external bottles with nutrient solutions (for the growing period, Table 2) connected over hoses and pipes to the plant pots in the labelboxes. Depending on the labelling regime the two boxes were supplied with different nutrient solutions 6b,c. The labelbox used for global ^15^N-labelling was supplied with salts enriched with ^15^N 6c. To prevent contamination of the air in the boxes during watering, the airspace in the nutrient solution bottles was connected with labelboxes to allow pressure compensation with air from inside the boxes.

**Table 2:** Adapted Hoagland solution

Salts	Hoagland Stock solutions [g/l]	Germination period [mg/l]	Growing period [mg/l]
KNO_3_^a^	202	101	404
Ca(NO_3_)_2 _× 4H_2_O^a^	236	472	472
C_10_H_12_N_2_NaFeO_8_ (Ferric sodium–EDTA)	15	3.75	0.975
MgSO_4 _× 7H_2_O	493	123.3	98.6
NH_4_NO_3_^a^	80	8	8
H_3_BO_3_	2.86	0.143	0.072
MnCl_2 _× 4H_2_O	1.18	0.59	1.18
ZnSO_4 _× 7H_2_O	0.22	0.88	0.33
CuSO_4_	0.051	0.051	0.051
Na_2_MoO_4 _× 2H_2_O	0.12	0.024	0.0072
KH_2_PO_4_	136	68	68

^a^Salts substituted with highly ^15^N-enriched analogues in the ^15^N-labellling experiment

#### CO_2_ scrubber

At the beginning of the cultivation, before placing the seedlings into the labelbox, CO_2_ was removed from air inside the labelbox. This is achieved by the use of CO_2_ scrubber which is attached to the gas hose between the membrane pump 5a and labelbox ①. It is filled with CO_2_ adsorbent (3–4 mm diameter, Soda Lime Carbon Dioxide adsorbent spherical granules, Spherasorb™, Intersurgical Ltd., Wokingham, UK) which traps CO_2_ after navigating the dry air from the labelbox through the CO_2_ scrubber. In the presented setup, switching of the valve to direct the gas flow through the CO_2_ scrubber has to be done manually.

### Chemicals

Methanol (MeOH, LC–MS CHROMASOLV^®^), acetonitrile (ACN, LC–MS CHROMASOLV^®^) and formic acid (FA, MS grade, ~ 98% purity) were purchased from Riedel-de Haën, Honeywell (Seelze, Germany). The ultra-pure water was obtained from an ELGA Purelab system Veolia Water (Ultra AN MK2, Vienna, Austria). The salts KOH (≥ 99.5%), NH_4_NO_3_ (≥ 99%), Na_2_MoO_4_*2H_2_O, KH_2_PO_4_ (≥ 99.8%), KNO_3_ (65%) were obtained from Merck (Darmstadt, Germany) and MgSO_4_*7H_2_O, ZnSO_4_*7H_2_O, Ca(NO_3_)_2_*4H_2_O, Ferric sodium - EDTA (C_10_H_12_N_2_NaFeO_8_), MnCl_2_*4H_2_O, ZnSO_4_*7H_2_O, CuSO_4_*5H_2_O (> 98%) from Sigma-Aldrich (Steinheim, Germany). NH_4_NO_3_ (^15^N, 98 atom %), Ca(NO_3_)_2_ (^15^N, 98 atom %), KNO_3_ (^15^N, 98 atom %) and ^13^CO_2_ (99% purity) was purchased from Eurisotop (St-Aubin, France) while CO_2_ and synthetic air were obtained from Messer (Gumpoldskirchen, Austria).

### Plant material

Wheat genotypes Karur (*T.durum*), Remus (*T.aestivum*) and Apogee (a dwarf cultivar of *T. aestivum*) were generated and used in native and labelled (^13^C and ^15^N) form. The native seeds of Karur and Remus were obtained from—and grown in the glasshouse at—the Institute of Biotechnology in Plant Production (University of Natural Resources and Life Sciences, Vienna, Department of Agrobiotechnology, IFA-Tulln, Austria). The native Apogee seeds were obtained from the Department of Applied Genetics and Cell Biology (University of Natural Resources and Life Sciences, Vienna, University Research Center Tulln, Austria) and grown in the labelbox while supplied with non-labelled substrates. The ^13^C- and ^15^N-labelled material of each genotype was produced in the labelbox from native seeds.

### Experimental description

The cultivation of *T.durum* plants lasted 87 days in total and comprised following steps: 1. preparation of the nutrient solution, wheat seedlings and labelboxes; 2. cultivation and 3. harvest.

### Preparation of the nutrient solution

To generate globally ^13^C- and ^15^N-labelled *T.durum* plants, the seedlings were grown in a perlite substrate using nutrient solutions adapted from Hoagland (1950) [45] and Bugbee, Spanarkel (1994) [46] (Table 2). The salt concentration was adapted to the growing stage of the plants resulting in two nutrient solutions (one for the germination and one for the growing period respectively, Table 2). Both labelling regimes were provided with the same nutrient solutions, with the only difference that the ^15^N-labelling experiment was carried out with highly ^15^N-enriched salts. From two stock solutions, a total of 4 different nutrient solutions were prepared for two developmental periods, i.e. 2 with labelled ^15^N (98–99 atom % enriched) and 2 with native ^14^N salts.

All 4 nutrient solution types were prepared from the Hoagland stock solutions (composition in Table 2) in cool (< 10 °C) ultra-pure autoclaved water and stirred for ca. 10 min on a magnet stirrer. Low amounts of ^12^C as well as ^14^N were present in the nutrient solutions to provide iron uptake in the form of ethylenediamine tetraacetic acid (EDTA) chelate complex.

### Preparation of wheat seedings

Before placement into the labelboxes, wheat seedlings were prepared according to the following three steps: germination, vernalisation and planting.

#### Germination

Wheat seeds were placed in blocks of rock wool (4 × 4 cm, Grodan) with the embryo upwards so that each block contained three seeds. The seed-contained blocks were placed in two separate darkly shaded boxes and watered with solutions for the germination period containing either ^15^N or ^14^N salts until saturation of the rock wool. Boxes were closed and the seeds germinated at room temperature in the darkness for 2 days.

#### Vernalisation

Germinated seedlings were proceeded to vernalisation in the dark for 2 days at 4 °C in the cooling room to promote shoot yield and acceleration of the flowering process [47]. Under these conditions, previous experiments have shown to result in 4–6 shoots per *T.durum* plant during cultivation.

#### Planting of seedlings

**Fig. 6:**
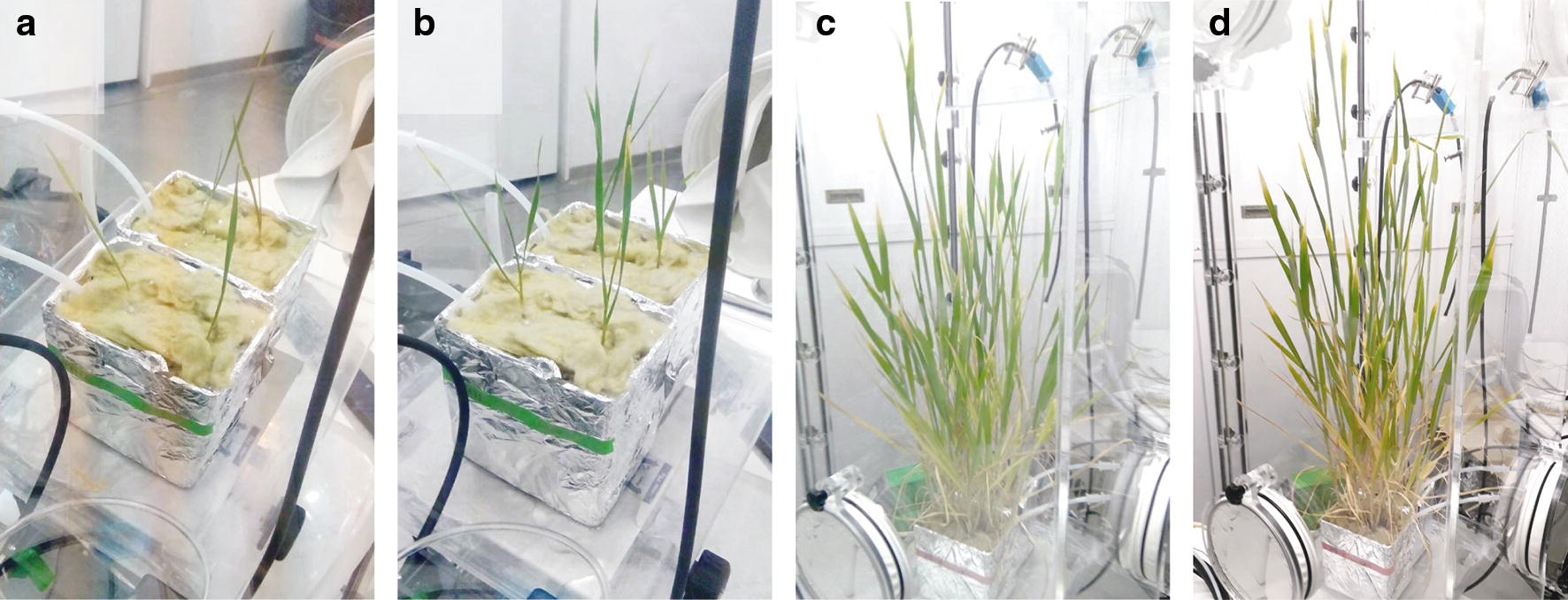
Wheat in the cultivation box at different growth stages: **a** seedling development (Z1), **b** tillering (Z2), **c** stem elongation (Z3), **d** heading (Z5)

Plant pots (~ 1 L) were wrapped in aluminum foil as depicted in Fig. 6, to prevent access of light to the medium and thus growth of algae. Pots were filled with perlite and nutrition solutions for the growth period (Table 2). For ^15^N-labelling nutrient solution containing ^15^N salts was used. Germinated and vernalised seedlings were transferred from rock wool blocks into corresponding prepared plant pots. After the placement of seedlings, a layer of rock wool was added on top of the pot, to reduce water evaporation from the nutrient solution and prevent access of light during plant cultivation.

### Preparation of the PhytolabelBox equipment

Prior to placement of plants into the labelboxes, the technical performance of the equipment was verified.

First, the tightness of labelboxes was tested. The labelboxes are operated with slight overpressure which is regulated at (10 ± 2) mbar above the ambient atmospheric pressure. The ability to keep the overpressure at the setpoint range can be taken as a measure of tightness of the system including the labelboxes. To check this, the pressure cycle consisting of the pressure drop from 10 mbar to 8 mbar and build up back to 10 mbar by dosage of synthetic air was monitored, resulting in pressure cycle periods of 10 min for the ^15^N- and 20 min for the ^13^C-labelbox respectively. These values slightly increased with the progress of the cultivation.

Second, the increase of CO_2_ in the labelbox per dosed loop was determined. This is required for the estimation of the CO_2_ consumption rate of plants after the cultivation is finished. Tests showed that per dosed loop of CO_2_, the concentration of CO_2_ in the labelbox increased by (4.6 ± 0.3) ppm ^12^CO_2_ in the ^15^N-labelbox and (9.7 ± 0.3) ppm in ^13^C-labelbox when the pressure reducing device on the CO_2_ bottles was set to 1 bar each.

### Cultivation of plants

Start of the cultivation.

1. The pots with seedlings were placed in the corresponding ^15^N- and ^13^C-labelboxes.
2. The labelbox cover at the back was tightly sealed with screws. Note: Too tightly sealed screws may damage the Plexiglas housing. To promote tightness a silicone paste was applied on the sealing gums of the labelboxes.
3. The atmospheric CO_2_ in the ^13^C-labelbox was removed by passing the air through the CO_2_ scrubber. Further, ^13^CO_2_ gas was manually dosed to reach a level of 400 ppm in the labelbox.
4. The setpoint values of CO_2_, O_2_, overpressure and humidity were defined in the software (Table 3).

**Table 3:** Setpoint values for the recorded and regulated atmospheric parameters for both labelboxes

Regulated parameters	Set point values	Allowed parameter range	
		Min.	Max.
CO_2_ [ppmv]	400	300	1600
O_2_ [%]	20	–	–
Overpressure [mbar]	10	8	12
Humidity [%]	Variable	–	70

CO_2_, O_2_ and overpressure were maintained at setpoints by the control unit while values below the minimum led to the activation of gas dosing system. The maximum allowed setpoint for relative humidity was defined while levels below depended on the plant biomass and thus was increasing with growth (see Fig. 1)
5. The cultivation was initiated by starting the measurement in the software. All user predefined setpoints were automatically monitored and regulated by the system during the whole cultivation period (Table 3).

### Cultivation conditions

The temperature and light-duration setpoints outside the labelbox for the day/night cycle were realised over an external control unit of the climate chamber, in which the labelboxes were located throughout the cultivation period. These setpoint values are shown in Table 4. The light intensity remained constant during the whole cultivation period. The light intensity at simulated day conditions was estimated with the Li-COR sensor (LI-190SA Quantum Sensor, Li-COR, Germany) and was measured on top of the rock wool immediately after placing the pots with seedlings into the labelbox to be 206 µmol/(m^2^·s) in the ^13^C-labelbox and 220 µmol/(m^2^·s) in ^15^N-labelbox respectively.

**Table 4:** Light and temperature intervals in the climate chamber during the cultivation process

Cultivation days	Light cycle day [h]/night [h]	Temperature day [°C]/night [°C]	Growth stage (Zadoks scale [48])
0–28	12/12	12/10	Z1 and Z2
28–31	12/12	14/12	Z2 and Z3
31–38	14/10	16/14	
38–86	14/10	18/16	Z3, Z4 and Z6

Zadoks scale: Z1– seedling development, Z2–tillering, Z3–stem elongation, Z4–booting and Z6–anthesis

### Irrigation

Irrigation rhythm was adapted to the growth stage of the plants. In the first 3 weeks of cultivation, the plants were irrigated once a week and from day 28 until harvest twice a week. Irrigation was accomplished manually by an external peristaltic pump (S20, Vederflex^®^ smart, UK) and a flow velocity of 100 ml/min. The volume of nutrient solution applied during watering was recorded. Both labelboxes were watered with the respective nutrient solution by the use of separate hoses.

### Harvest

Each ear and the adjacent stems, leaves and roots inside the ^13^C-labelbox were harvested separately 144 h after the first anthers had appeared on the ear, respectively. The sample material was removed via the gate compartment with an air lock system and immediately shock frozen in liquid nitrogen outside the labelboxes. The time between cutting the samples and freezing was kept as short as possible. The ^15^N-plants were harvested under similar experimental conditions while the ^15^N-labelbox was open, as the ambient atmosphere does not disrupt the ^15^N-labelling process.

### Freeze-drying and sample storage

Sample material was freeze-dried (FreeZone 6Plus, Labconco, Kansas City, MO, USA) to < 5% rest moisture for long term storage at − 80 °C. To this end, wheat ears were dried for 6 days, stems for 1 day and leaves for 2 days at − 80 °C and ~ 0.4 mbar. Rest moisture was estimated with the infrared moisture analyzer (LC 4800P-OOV1, Sartorius, Göttingen, Germany).

### Sample preparation

Two set of samples were prepared for the measurement. One is used for the determination of the degree of enrichment and the other for the annotation of the global C and N metabolome. The procedure for milling and extraction as well as the solvent composition of samples at the time point of measurement was the same in both cases. The only difference is in the composition of the sample extracts. For annotation of the global metabolome, wheat ear extracts of freshly sampled native wheat from glasshouse were mixed with either freeze-dried ^12^C^15^N or freeze-dried ^13^C^14^N ears from the labelbox. For the determination of the degree of enrichment, extracts of either native, ^12^C^15^N- or ^13^C^14^N- wheat ears were used.

The wheat ears were milled to a fine powder with a ball mill (MM400, Retsch, Haan, Germany) while being kept in frozen condition. The extraction was performed similarly as reported in [8]. In short, 100 mg of fresh native wheat powder and 30 mg of dried wheat powder was extracted separately with 1 ml MeOH/ACN/H_2_O (1.5/1.5/1 v/v/v) + 0.1% formic acid (FA). 70 µl of H_2_O was added to the extraction solvent of dried wheat in order to compensate for the loss of water during the drying process. For the detection of the global C and N metabolomes, native extracts were mixed 1:1 (v/v) with ^13^C^14^N or ^12^C^15^N extracts and diluted with H_2_O + 0.1% FA in order to obtain 1:1 organic: water ratio prior to LC-HRMS [8]. For determination of the degree of enrichment, the labelled and native extracts were diluted with H_2_O + 0.1% FA individually.

### LC-HRMS measurement

The LC-HRMS measurement was performed in positive ionisation mode on an Orbitrap mass spectrometer (QExactiveHF, Thermo Fisher Scientific, Bremen, Germany) coupled to a Vanquish uHPLC (Thermo Fisher Scientific, Bremen, Germany) with the method described in [49].

### Data processing

Data processing was performed with the AllExtract module in MetExtract II software [14]. Here, only LC-HRMS spectra of the sample set for global C and N metabolome annotation were considered. Briefly summarised, the MetExtract II software was set up to search for pairs of chromatographic peaks, which originate from co-eluting native and (^13^C- or ^15^N-) labelled metabolite ion forms. Native and labelled metabolite forms must show their distinctive and mirror-symmetric isotopologue patterns. Moreover, their chromatographic peak shapes must be highly similar and show perfect coelution. Such detected metabolite ions were then aligned across all samples of the either ^13^C- or ^15^N-labelling experiment.

The measured LC-HRMS raw files were converted into mzXML format using the MSConvertGUI (version 3.0.19166-cc86d1f56) from Proteowizard [50], and further loaded in the MetExtract II. Data processing parameters of MetExtract II were: Intensity threshold for M and M’: 10,000 counts; Chromatography start and end time: 3–36 min; Chromatographic peak scales: 7–21; Maximum allowed deviation for signal pairs: ± 3 ppm; Maximum isotopologue abundance error: ± 15%; Minimum peak correlation: 0.85 (Pearson correlation); MZ-delta between native and labelled metabolite ion form: 1.00335484 for the ^13^C-labelling experiment and 0.99703000 for the ^15^N-labelling experiment; Isotopologue purity: 0.9893 for the native metabolite form, 0.9850 for the ^13^C-labelled metabolite ion form, 0.9951 for the ^15^N-labelled metabolite ion form; Maximum MZ deviation of consecutive signal pairs: ± 8 ppm; Number of isotopologues checked: 2; Number of carbon atoms searched for in the ^13^C-labelling experiment: 3–60; Number of nitrogen atoms searched for in the ^15^N-labelling experiment: 1–12. Parameters for the combination of the ^13^C- and the ^15^N-labelling experiment: Maximum allowed MZ deviation: 5 ppm; Maximum allowed retention time shift: ± 0.15 min.

## Supplementary information

**Additional file 1:** Calculation of the CO_2_ consumption.

## Data Availability

The datasets used and/or analysed during the current study are available from the corresponding author on reasonable request.

## Abbreviations

^13^C: Stable isotope of carbon containing 6 protons and 7 neutrons
^13^C-labelbox: Cultivation chamber for ^13^C-labelling
^15^N: Heavy stable isotope of nitrogen which contains 7 protons and 8 neutrons
^15^N-labelbox: Cultivation chamber for ^15^N-labelling
DW: Dry weight
FW: Fresh weight
GC-MS: Gas chromatography-mass spectrometry
Labelbox: Cultivation chamber in which stable isotopic labelling is performed
LC-HRMS: Liquid chromatography-high resolution mass spectrometry
MS: Mass spectrometry
mzXML: File format for LC-HRMS data
NMR: Nuclear magnetic resonance
*T. aestivum*: *Triticum aestivum*
*T. durum*: *Triticum durum*
Z1: Growth stage of wheat after Zadoks scale [48] and represents seedling development
Z2: Growth stage of wheat after Zadoks scale [48] and represents tillering
Z3: Growth stage of wheat after Zadoks scale [48] and represents stem elongation
Z4: Growth stage of wheat after Zadoks scale [48] and represents booting
Z5: Growth stage of wheat after Zadoks scale [48] and represents heading
Z6: Growth stage of wheat after Zadoks scale [48] and represents anthesis
