# Supplementary material for: Preparation of uniformly labelled 13C- and 15N-plants using customised growth chambers
Source: Plant Methods. 2020 Apr 6;16:46. doi: 10.1186/s13007-020-00590-9 (PMC7137243; doi:10.1186/s13007-020-00590-9)
Supplement: Supplementary file 1 — Additional file 1: Calculation of the CO2 consumption. [file 13007_2020_590_MOESM1_ESM.docx]

# Additional file

## Additional file 1

### Calculation of the CO_2_ consumption

To determine the ^13^CO_2(g)_ consumption rate, the consumed CO_2_ was calculated in volume units (V_CO2_) with **equation (S1)** and in molar units (n_CO2_) with **equation (S4)**. The **equation (S4)** was derived from the Law of Avogadro’s **(equation S3**) representing the concertation in ppm by volume and **equation (S3)** is the. In **equation (S1)** included is: the total count of introduced loops of CO_2(g)_ into the labelbox (X_dosed loops_) during the cultivation; the experimentally determined increase of CO_2_ concertation per loop dosage (c_CO2 per loop_) and the total volume of the labelbox (V_LB_). Further, in the **equation (S4)**, under V_m_ considered is the standard volume of the real gas (V_m_ =24 L).

| $\boldsymbol{V}_{\boldsymbol{CO}\boldsymbol{2}}\left[ \boldsymbol{l} \right]\boldsymbol{=}\boldsymbol{c}_{\boldsymbol{CO}_{\boldsymbol{2}}\boldsymbol{per loop}}\left[ \boldsymbol{ppm} \right]\boldsymbol{*}\boldsymbol{x}_{\boldsymbol{dozed loops}}\boldsymbol{*}\boldsymbol{V}_{\boldsymbol{LB}}\boldsymbol{[l]*}\boldsymbol{10}^{\boldsymbol{6}}$ | (S1) |
| --- | --- |
| $c_{{CO}_{2}}\left[ ppmv \right] = \frac{V_{CO2}[\mu l]}{V_{LB}[l]}$ | (S2) |
| $V_{m}[l/{mol}]=\frac{V_{CO2}[l]}{n_{CO2}[mol]}$ | (S3) |
| $\boldsymbol{n}_{\boldsymbol{CO}\boldsymbol{2}}\left[ \boldsymbol{mol} \right]\boldsymbol{=}\boldsymbol{c}_{\boldsymbol{CO}\boldsymbol{2}}\left[ \boldsymbol{ppmv} \right]\frac{\boldsymbol{V}_{\boldsymbol{LB}}\left[ \boldsymbol{l} \right]}{\boldsymbol{V}_{\boldsymbol{m}}\left[ \boldsymbol{l} \right]}\boldsymbol{*}\boldsymbol{10}^{\boldsymbol{-}\boldsymbol{6}}$ **(S2) + (S3)** | (S4) |
